# Cancer: New Chlorpyrifos Link?

**Published:** 2005-03

**Authors:** Julian Josephson

Chlorpyrifos, one of the most widely used organophosphate insecticides in the United States, is a known neurotoxicant, but is it carcinogenic too? A team of National Cancer Institute (NCI) researchers recently reported on the first epidemiologic study to carefully evaluate cancer among chlorpyrifos applicators. Their results suggest a possible link between the insecticide and lung cancer.

Chlorpyrifos’s neurotoxicity led to its 2000 ban from numerous residential uses, including termite suppression and use in pet collars. Nevertheless, it is still widely used for many nonresidential purposes, including pest control for turf and ornamental plants. It may be used indoors in warehouses, ship holds, boxcars, factories, and food processing plants, or as containerized pesticide baits in child-resistant packages. Currently, about 800 registered products contain this compound.

The current evaluation was done as a part of the Agricultural Health Study, which is supported by the NCI, the NIEHS, and the Environmental Protection Agency (EPA), in collaboration with the University of Iowa and Battelle. Won Jin Lee, a visiting fellow in the NCI Occupational and Environmental Epidemiology Branch, and his team evaluated the incidence of cancer among a cohort of 54,383 licensed pesticide applicators in Iowa and North Carolina. They assessed the association between chlorpyrifos exposure and cancer incidence after adjusting for known or suspected confounding factors. Study subjects were recruited between 1993 and 1997 and followed for an average of 6.4 years.

About 3.8% of the applicators developed malignant lung neoplasms. The researchers found a statistically significant exposure–response relationship between increasing chlorpyrifos exposure and lung cancer, but not for any other cancer examined. The most frequent users of chlorpyrifos had a relative risk of lung cancer of about twice that of nonusers, an association that could not be explained by smoking, previous lung disease, other occupational exposures, or type of farming operation. The results were published in the 1 December 2004 issue of the *Journal of the National Cancer Institute*.

Study coauthor Aaron Blair, a senior investigator in the Occupational and Environmental Epidemiology Branch, says, “The elevated risk of lung cancer observed must be balanced against the general lack of evidence for [chlorpyrifos’s] carcinogenicity from experimental investigations. This issue will be further evaluated in a future follow-up of the Agricultural Health Study cohort.”

Debra Edwards, director of the Special Review and Reregistration Division in the EPA Office of Pesticides, says the EPA agrees that the new study must be considered along with all the available animal studies showing that chlorpyrifos does not cause cancer. She says, “[The EPA] will continue to work with the NCI and others as the postulated link beween chlorpyrifos and lung cancer is sorted out.

